# Native Mass Spectrometry‐Guided Screening Identifies Hit Fragments for HOP‐HSP90 PPI Inhibition**

**DOI:** 10.1002/cbic.202200322

**Published:** 2022-09-20

**Authors:** Michaelone C. Vaaltyn, Maria Mateos‐Jimenez, Ronel Müller, C. Logan Mackay, Adrienne L. Edkins, David J. Clarke, Clinton G. L. Veale

**Affiliations:** ^1^ The Biomedical Biotechnology Research Unit (BioBRU) Department of Biochemistry and Microbiology Department Rhodes University Makhanda 6139 South Africa; ^2^ EaStCHEM School of Chemistry Joseph Black Building, David Brewster Road Edinburgh EH93FJ UK; ^3^ School of Chemistry and Physics University of KwaZulu-Natal Scottsville 3209 South Africa; ^4^ Department of Chemistry University of Cape Town Rondebosch Cape Town 7700 South Africa

**Keywords:** fragment-based drug discovery, native mass spectrometry, PPI inhibitors, heat shock protein 90, HSP70-HSP90 organizing protein

## Abstract

Contemporary medicinal chemistry considers fragment‐based drug discovery (FBDD) and inhibition of protein‐protein interactions (PPI) as important means of expanding the volume of druggable chemical space. However, the ability to robustly identify valid fragments and PPI inhibitors is an enormous challenge, requiring the application of sensitive biophysical methodology. Accordingly, in this study, we exploited the speed and sensitivity of nanoelectrospray (nano‐ESI) native mass spectrometry to identify a small collection of fragments which bind to the TPR2AB domain of HOP. Follow‐up biophysical assessment of a small selection of binding fragments confirmed binding to the single TPR2A domain, and that this binding translated into PPI inhibitory activity between TPR2A and the HSP90 *C*‐terminal domain. An *in‐silico* assessment of binding fragments at the PPI interfacial region, provided valuable structural insight for future fragment elaboration strategies, including the identification of losartan as a weak, albeit dose‐dependent inhibitor of the target PPI.

## Graphical abstract

**We sought to** demonstrate the combined utility of native mass spectrometry (MS), and fragment‐based drug discovery as a viable means for identifying protein‐protein interaction (PPI) inhibitors. Here we identified a small cohort of molecular fragments, who's binding to the TPR2AB region of our target, HOP, translated into inhibition of the PPI between the TPR2A domain of HOP and the HSP90 *C*‐terminus.

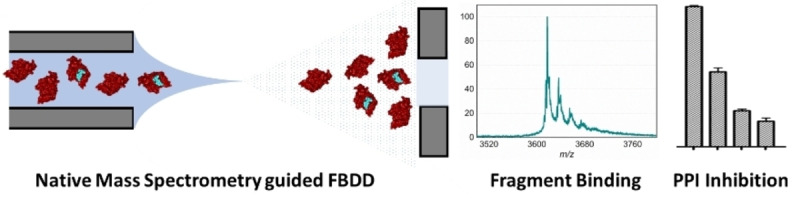

## Introduction

Interactions between proteins are key regulators of cellular biology and provide opportunities to expand druggable chemical space.^[1]^ However, in contrast to classical enzyme and receptor targets, the interfaces between proteins are comparatively featureless, lacking the molecular topography commonly associated with small molecule binding.[^2^, ^3^] Additionally, protein interfaces are not associated with endogenous ligands,^[2]^ which creates a significant challenge for conventional approaches to drug discovery. While protein‐protein interactions (PPIs) have traditionally been considered difficult to drug, rapid growth in the application of biophysical methodology to inhibitor screening has not only facilitated the characterization of PPI interfaces but has also made it possible to detect binding events between biomolecules and weakly interacting small molecules, thus ushering in the era of fragment‐based drug discovery (FBDD).[^4^, ^5^] The enhanced efficiency with which chemical space can be sampled in the absence of redundant chemical functionality is critical when considering the relatively featureless interface of a PPI.^[6]^

Whilst undeniably successful, the most commonly accessed biophysical methods for fragment screening, which include NMR, X‐ray co‐crystallography, ITC and SPR, have drawbacks associated with sample consumption, sensitivity and experimental set up.^[7]^ Whilst conventionally underutilised, the speed and sensitivity inherent to native mass spectrometry (MS) has seen it emerge as a powerful fragment screening technique capable of detecting weak interactions between unlabelled and untethered small molecular fragments and target proteins in the gas phase.[^8^, ^9^, ^10^, ^11^] MS ligand binding experiments typically look to observe a molecular ion correlating to the apo protein in its native state (M) with the emergence of a new ion corresponding to the deconvoluted molecular mass of the protein‐ligand complex (M**⋅**L), indicating ligand binding. Comparison of the relative intensities of these peaks provides a rough indication of binding strength.^[12]^ This comparatively simple and fast means of interpreting ligand binding makes native MS particularly suited to the screening portion of an FBDD campaign. While entropic contributions to binding free energy, such as hydrophobic interactions are weakened in the gas phase, enthalpic contributions to binding, which typically involve interactions between polar functional groups, survive the transmission into the gas phase.^[13]^ As such, native MS screening will tend to favour compounds with preferable solubility and polarity characteristics, thus improving the likelihood of retaining suitable physicochemical properties following entropy‐governed hit/lead optimization campaigns.^[14]^ However, despite this promise, literature evidence of the use of native MS FBDD for identifying PPI inhibitors is extremely limited.^[15]^

Our target in this study, the HSP70‐HSP90 organizing protein (HOP), is a co‐chaperone which binds simultaneously to HSP70 and HSP90, acting as a scaffold for the transfer of partially folded client proteins between the two. Accordingly, HOP couples the *de novo* and stress‐related protein folding pathways of HSP70 to the conformational regulation cycle of HSP90.^[16]^

Furthermore, formation of the HSP70‐HOP‐HSP90 ternary complex is required for proteasome assembly and therefore influences the efficiency of proteasomal‐mediated protein turnover.^[17]^ HSP90 is highly conserved and structurally indistinguishable whether found in normal or tumour cells. In normal cells, HSP90 is commonly found in its latent uncomplexed form. In contrast, in the tumour environment, HSP90 is dependent on the presence of co‐chaperones to facilitate the rapid production of primary metabolites. As such, it is found predominantly in high molecular weight activated complexes with co‐chaperones, such as HOP, that facilitate malignancy.^[18]^ Therefore, inhibition of the HSP90‐HOP complex has been postulated as a selective anticancer target.[^19^, ^20^]

**Figure 1:**
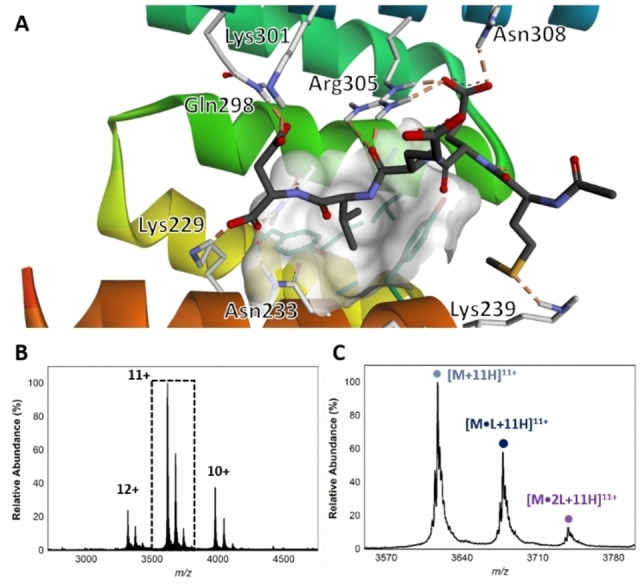
A. X‐ray co‐crystal of Ac−MEEVD−OH (**1**) bound to the TPR2A domain of HOP (PDB 1ELR). Glu2 and Asp5 form a series of salt bridges with Lys229, Asn233, Gln298, Lys301, Arg305 and Asn308 residues, while Val4 occupies a hydrophobic pocket (white surface) lined with aromatic residues Tyr236 and Tyr248. B. Native mass spectrum of the 1 : 1 buffered solution of TPR2AB and **1**. C. Expanded region of the 11^+^ charge state, showing the apo TPR2AB (*m/z* 3618.8, [M+11H]^11+^) the TPR2AB‐**1** complex (*m/z* 3679.1, [M**⋅**1 L+11H]^11+^) and TPR2AB bound to two Ac−MEEVD−OH peptides (*m/z* 3739.5, [M**⋅**2 L+11H]^11+^).

The primary HOP‐HSP90 binding interface is mediated by an interaction between the acid‐rich *C*‐terminal MEEVD motif of HSP90 and the ‘carboxylate clamp’ region of the TPR2A domain of HOP, which features a series of basic amino acid residues. X‐ray co‐crystallisation of *N*‐acetylated MEEVD (Ac−MEEVD−OH, **1**) with TPR2A (PDB 1ELR) revealed a network of salt bridges formed between Glu2 and Asp5 of **1**, with the carboxylate clamp (Figure 1A). Furthermore, Val4 was found to occupy a shallow hydrophobic pocket.^[21]^

Kawakami and co‐workers exploited these structural features to develop a hybrid TPR peptide designed to interact with the acidic MEEVD region of HSP90 and block its pro‐oncogenic interaction with the TPR2A domain of HOP.^[22]^ This idea was expanded by McAlpine and co‐workers, who reported a series of TPR‐inspired cyclic peptides whose HSP90 interaction disrupted PPI formation and HSP90’s folding function.^[23]^ Pimienta et al. (**2**), followed more recently by Darby et al. (**3**), investigated the opposite face of the PPI, identifying small molecules which bound to TPR2A and disrupted binding of *C*‐terminal MEEVD containing peptides without explicitly demonstrating PPI inhibition.[^24^, ^25^] Given the potential of this target, we have also investigated inhibitors of this PPI, resulting in the identification of tetrazole peptide (**4**), whose binding to TPR2A resulted in disruption of the TPR2A‐HSP90 PPI in the sub‐micromolar range.^[26]^

Given the efficiency of FBDD for probing chemical space, as well as the aforementioned advantages of native MS, we reasoned that a nano‐electrospray ionisation (nano‐ESI) native MS fragment screening workflow might simultaneously demonstrate the utility of this technique for general PPI drug discovery, whilst providing a fruitful means of identifying HOP‐HSP90 PPI fragment hits. Furthermore, the preferable physicochemical properties of MS derived hits would facilitate elaboration into new classes of HOP‐HSP90 PPI inhibitors, with potential for disrupting cellular proteostasis and ultimately new cancer therapeutics.


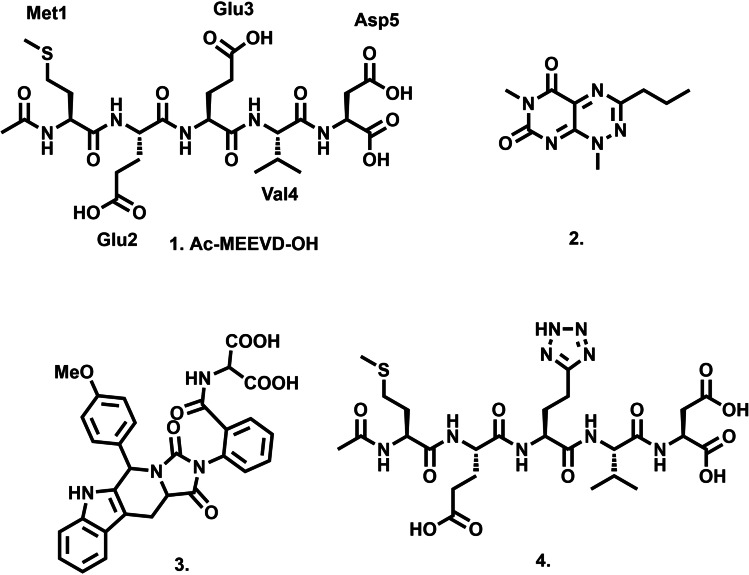


## Results and Discussion

With the aim of targeting the basic residues of the carboxylate clamp region of TPR2A, we assembled a ‘cherry‐picked’ fragment library of 133 compounds, which all contained either a carboxylic acid or tetrazole functionality. TPR2A exists as a single folding unit with the TPR2B domain of HOP and as such imparts additional stability to the protein when compared to TPR2A alone. Accordingly, considering the high fragment concentrations typically required for FBDD screening and the possibility of protein destabilisation, we opted to conduct our screen against the combined TPR2AB domains.

The TPR2B domain of HOP features its own carboxylate binding region. However, several studies have shown that this region displays substantially weaker affinity for both the *C*‐terminal region of HSP90 and the MEEVD pentapeptide compared to TPR2A. Furthermore, binding affinities between the HSP90 *C*‐terminus with either TPR2A or combined TPR2AB are indistinguishable, indicating that TPR2B has a negligible effect on the MEEVD‐TPR2A interaction.[^27^, ^28^, ^29^] Nonetheless, the presence of an additional carboxylate binding region presented a potential region for additional fragment binding and thus a possible challenge for comparing the relative abundance of apo TPR2A and fragment bound species. However, for an initial screen, the stability imparted by the single fold of TPR2AB in the presence of comparatively high fragment concentrations was considered a suitable compromise. After initial triaging of the library, hits of interest could be further validated against the single domain. As a means of developing our native MS experimental conditions, we first looked to observe the gas phase association between the synthetic Ac−MEEVD−OH peptide and TPR2AB. TPR2AB and Ac−MEEVD−OH were incubated at a relative concentration of 1 : 1 (both 6 μM), whereat the major charge state (11^+^), we observed the apo TPR2AB (M) species, alongside the TPR2AB−Ac−MEEVD−OH (M**⋅**L) complex at a binding ratio of approximately 1 : 0.6 (Figure 1B and C), The presence of both an apo and Ac−MEEVD−OH bound species was entirely consistent with previous reports.^[26]^ Given the reported K_d_ by SPR of the Ac−MEEVD−OH−TPR2A interaction of 11 μM,^[27]^ the relative abundance of Ac−MEEVD−OH binding by native MS at 6 μM was considered satisfactory. In addition, we observed the formation of a minor (M**⋅**2 L), corresponding to the binding of two Ac−MEEVD−OH peptides, possibly due to minor binding to TPR2B.

**Figure 2:**
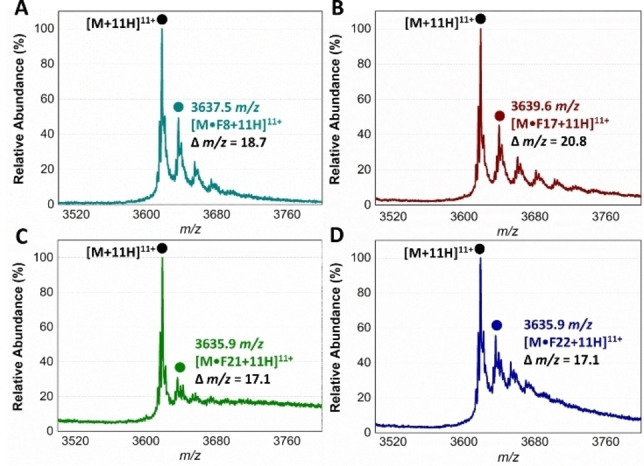
Expanded region of the 11^+^ charge state of a buffered mixture of TPR2AB with four selected binding fragments, **F8** (A), **F17** (B), **F21** (C) and **F 22** (D). Binding ratio was estimated based on the relative abundance of apo TPR2AB (*m/z* 3618.8, [M+11H]^11+^) and TPR2AB‐fragment complex and shown in Tables 1 and 2. Fragment binding was confirmed based on the Δ *m/z* from the apo TPR2AB peak.

Using this technique to demonstrate the ability to characterize noncovalent protein: ligand interactions, we then turned our attention to fragment screening by native MS. Fragment screening was conducted at a final protein: fragment concentration of 6 μM: 250 μM. Each fragment was assessed following the summing of scans for four minutes. To avoid over‐estimating fragment binding, binding affinity was evaluated and ranked only by the relative intensity of the M**⋅**L peak compared to the apo M peak (Figure 2).

Calculating the expected Δ *m/z* confirmed that the M**⋅**L peak was due to fragment binding. For example, **F8** (MW=206.12 Da) would result in a Δ *m/z* of 18.7 at the 11^+^ charge state and a peak of *m/z* 3637.5 [M**⋅F8**+11H]^11+^. This assessment indicated that 29 fragments in the library bound to TPR2AB (Table 1 and 2), while 104 fragments (78 %) did not form an (M**⋅**L) peak (**S1**–**S104**, Table S1).

**Table 1:** Structures, expected Δ *m/z* and binding ratios of Group 1 binding fragments.

	Structure	Δ *m/z*	M : M**⋅**L			Structure	Δ *m/z*	M : M**⋅**L
**F1**	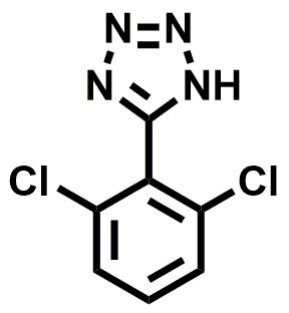	19.5	1 : 0.50		**F9**	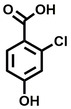	15.7	1 : 0.65
**F2**	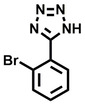	20.5	1 : 0.50		**F10**	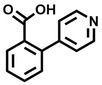	18.1	1 : 0.25
**F3**	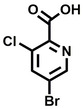	21.5	1 : 0.50		**F11**	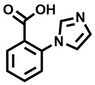	17.1	1 : 0.25
**F4**	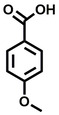	13.8	1 : 0.45		**F12**	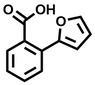	17.1	1 : 0.40
**F5**	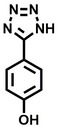	14.7	1 : 0.80		**F13**	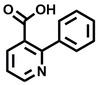	18.1	1 : 0.45
**F6**	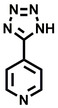	13.4	1 : 0.65		**F14**	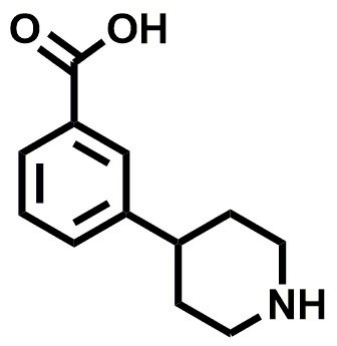	18.6	1 : 0.25
**F7**	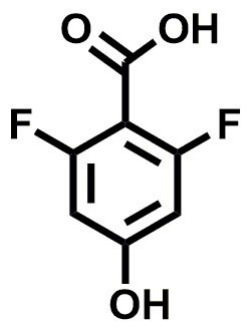	15.8	1 : 0.45		**F15**	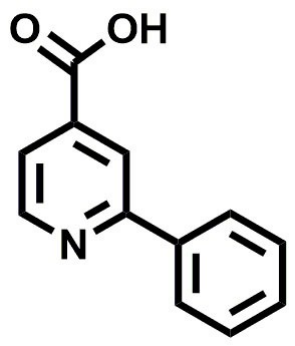	18.1	1 : 0.45
**F8**	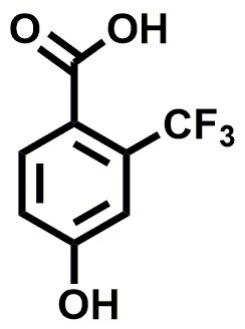	18.7	1 : 0.5					

**Table 2:** Structures, expected Δ m/z and binding ratios of Group 2 and 3 binding fragments.

	Group 2	Δ *m/z*	M : M**⋅**L		Group 3	Δ *m/z*	M : M**⋅**L	
**F16**	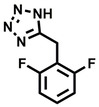	17.8	1 : 0.55		**F25**	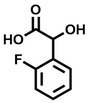	16.0	1 : 0.60
**F17**	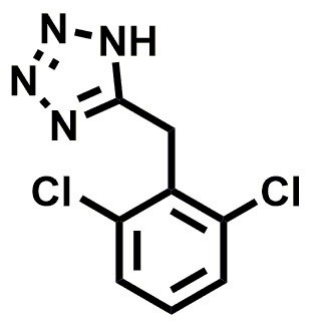	20.8	1 : 0.40		**F26**	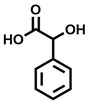	18.7	1 : 0.15
**F18**	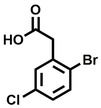	22.7	1 : 0.40		**F27**	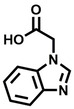	18.7	1 : 0.55
**F19**	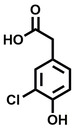	16.9	1 : 0.50		**F28**	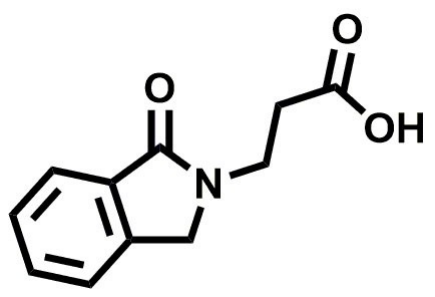	19.9	1 : 0.60
**F20**	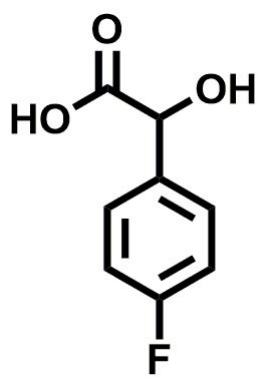	15.5	1 : 0.55		**F29**	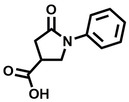	19.7	1 : 0.40
**F21**	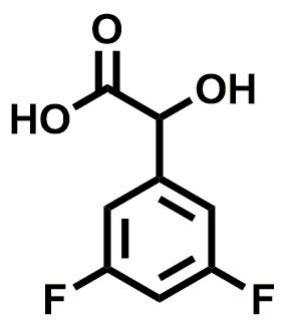	17.1	1 : 0.30					
**F22**	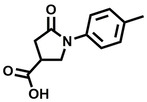	17.1	1 : 0.55					
**F23**	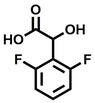	15.5	1 : 0.40					
**F24**	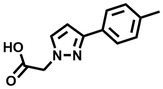	13.8	1 : 0.60					

Apart from providing insight into some structural requirements for binding, non‐binding fragments also demonstrated that the MS methodology did not inherently promote non‐specific adducting, which given the high fragment concentrations and high surface contact area inherent to PPI interfaces, was an initial concern. Binding was generally accompanied by additional multiple binding events (e. g. M**⋅**2 L, M**⋅**3 L) in the mass spectra. This phenomenon is common at the high concentrations used for fragment screening, although not always observable using many fragment screening techniques.[^30^, ^31^] Furthermore, the relatively topologically featureless PPI interface of TPR2A has several potential regions where fragments could interact simultaneously, with varying degrees of binding affinity. Accordingly, given the absence of binding for the majority of the library, we were confident that this observation was not a result of nonspecific adducting but rather a result of multiple interactions with our target protein.

An initial assessment of the screening results revealed that only fragments featuring an aromatic moiety were found to bind, with the majority of these possessing a six‐membered aromatic ring attached either directly to the acidic functional group or through a benzylic carbon. With a few exceptions, it was generally observed that fragments possessing a five membered aromatic ring attached directly to the acidic group, fragments possessing fused rings, and fragments featuring acidic side chains longer than two carbons tended not to bind (Table S1). Since MEEVD features two long chain glutamic acids residues and no aromatics, these results were initially surprising. However, the hydrophobic Val4 binding site on TPR2A is lined by two tyrosine residues (Tyr236 and Tyr248), while a third aromatic residue (Phe270) is located near the Glu2 binding region (Figure 1A).

Accordingly, we postulate that whilst in solution, these aromatic residues interact with aromatic fragments, thus facilitating salt‐bridge formation, which is maintained into the gas phase. Based on their rudimentary structural characteristics, the binding fragments were clustered into three groups, namely the benzoic acid analogues (**F1**–**F15** including pyridine‐containing analogues, Table 1), phenyl acetic acid analogues (**F16**–**F24**, Table 2), and a third group (**F25**–**F29**, Table 2) containing a mix of structural motifs, not fitting into groups 1 and 2.

Group 1 contained four fragments in which a tetrazole ring acted as the acidic principle. Amongst these, **F1** and **F2** were decorated with dual or single halogen substituents *ortho* to the tetrazole. In the case of fragments **F5** and **F6**, the tetrazole ring was attached to a phenol or pyridyl ring respectively, with the additional heteroatoms positioned para to the tetrazole. Importantly, fragment **F5** was particularly prone to multiple binding events. In agreement with these results, carboxylic acid containing fragments **F7**–**F9** contained both the *ortho* halogen and 4‐hydroxyl motifs, while fragments **F10**–**F12** contained a larger aromatic substituent at the *ortho* position without a 4‐hydroxyl.

Interestingly, fragments **S48** and **S63**, which are carboxylic acid containing analogues of **F2**, as well as the methoxy derivative (**S68**), did not bind, which in comparison to the binding fragments suggests that the tetrazole is preferred for binding. Furthermore, a para‐hydroxyl or possibly a corresponding pyridine moiety enhances fragment binding. While further, in the context of **F10**–**F12**, the chemical properties of the *ortho* substituent play an important role in fragment binding, including possibly their steric bulk and ability to disrupt the planarity between the phenyl ring and acid principle.

Similarly, while both the picolinic (**F3**) and nicotinic (**F13**) acid analogues, which also featured either a halogen or phenyl substituents *ortho* to the acid moiety, were found to bind, the corresponding *ortho* methyl (**S26** and **S16**) containing analogues, did not bind. While in general, an M**⋅**L peak was not detected for fragments featuring substituents *meta* to the acidic principle, two benzoic acid fragments (**F14** and **F15**) were identified as TPR2AB binders. While fragment **F14** was a comparatively weak binder, the piperidine moiety made it unique amongst the binding fragments. As highlighted earlier, a *para* hydroxyl substituent was seemingly beneficial for fragment binding.

While binding was also observed for methoxy analogue **F4**, most fragments featuring larger substituents in this position (e. g. **S4**, **S5**, **S52**, **S102**) did not bind, indicating a possible size restriction at this position. The patterns observed amongst group 2 binders largely mirrored the patterns observed for group 1, including a preference for halogens substituted *ortho* to the benzylic position (e. g. **F16**, **F17**, **F18**) or a para hydroxyl substituent (**F19**). Amongst this subset of fragments, a series of mandelic acid analogues (**F20**–**F24**) emerged as prominent binders, with the alpha hydroxyl moiety presumably playing an important role, with the effect of its stereochemistry currently unknown. While comparisons of the binding data for **F22**–**F24** make it unclear whether *ortho* halogens enhanced binding, the binding ratio of **F21** suggested that *meta* substitution also disrupted binding to some extent. While no distinct patterns could be drawn from group 3, **F26**–**F28** all contained a gamma lactam and were, in general, closer representatives of a peptidomimetic small molecule. Most importantly, **F25**–**F29** were structurally distinct from the fragments in groups 1 and 2 and possibly occupied different space at the PPI interface, thus representing opportunity for fragment elaboration or tethering.

**Figure 3:**
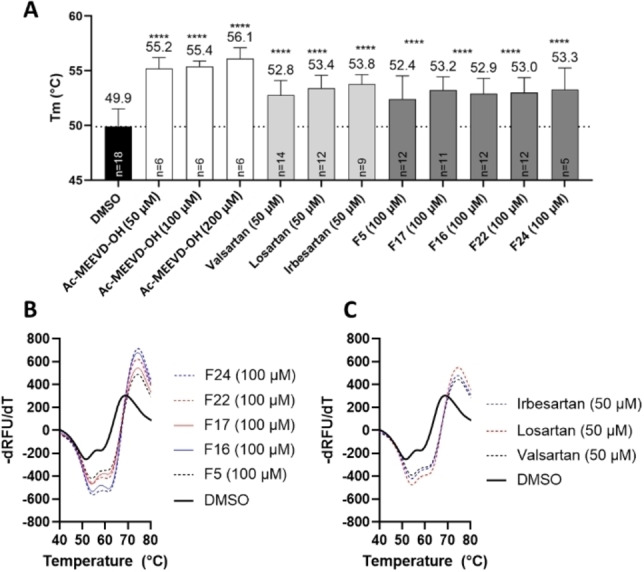
A. Thermal shift assay demonstrating ligand‐induced stabilisation of TPR2A. Data shown are averages ±SD. The average melting temperature (Tm) is shown above the bars. **** p<.0001 comparing TPR2A Tm in the presence of vehicle control (DMSO) to Tm in presence of ligands by one‐way ANOVA. Melt curves for the TPR2A protein in the absence (solid black line) and presence (dashed or coloured lines) of B fragments or C ‘sartan’ drugs. The shift in the location of the highest negative peak to a higher temperature indicates stabilisation of the protein in the presence of ligands.

To further narrow down whether our fragments bound to the target TPR2A single domain, a small subset of five promising fragments (**F5**, **F16**, **F17**, **F22** and **F24**) were selected for orthogonal thermal shift assay (TSA) using the single TPR2A domain of HOP. Control experiments showed a statistically significant shift in thermal stability in the presence of synthetic Ac−MEEVD−OH, when compared to DMSO (Figures S1 and 3 A). Similarly, the presence of the five selected fragments resulted in a statically significant, albeit less pronounced shift in thermal stability, indicating that in accordance with the native MS data, all five fragments bound the target protein, whilst further showing that some of these binding events occurred at TPR2A (Figure 3 A and B).

**Figure 4:**
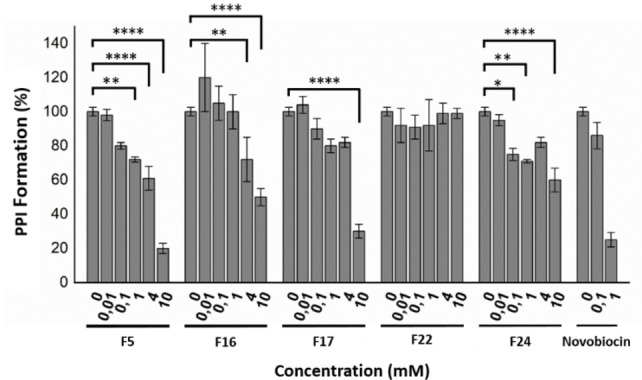
Solid phase PPI ELISA assay, between the HOP TPR2A domain and the HSP90 *C*‐terminal domain, in the presence of several selected fragments. **F5**, **F16** and **F17** displayed the most promising dose‐dependent PPI inhibition. * p<.05; ** p<.01; *** p<.001; **** p<.0001.

Having confirmed binding to TPR2A, these same five fragments were subjected to an ELISA solid phase PPI inhibitory assay (Figure 4), between the single TPR2A domain of HOP and the *C*‐terminal HSP90 domain.

The most effective PPI inhibitors (**F5**, **F16** and **F17**) showed a dose‐dependent inhibition of the target PPI at concentrations typically utilised in early FBDD. Importantly, these data confirmed that the binding observed in the MS screen was, at least partially, a result of TPR2A binding in a region required for PPI formation. While **F24** showed some promising, albeit substantially reduced PPI inhibitory activity, its *ortho* difluoro analogue (**F22**) was inactive in this assay, suggesting that this fragment primarily binds in a region of TPR2A which does not impact HSP90 PPI formation, as well as possibly the carboxylate clamp of TPR2B.

Having demonstrated promising PPI inhibition, we turned our attention to possible fragment elaboration strategies.

To that end, we examined the predicted binding modes of our binding fragments *in‐silico* using a previously reported method.^[26]^

**Figure 5:**
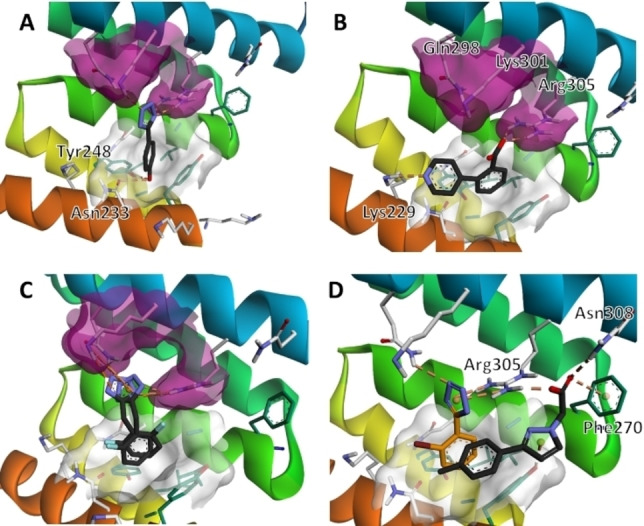
A–C. Docked binding pose of **F5**, **F10** and **F16**, respectively. Group 1 and 2 fragments were predicted to bind at the hydrophobic Val4 binding pocket (white surface), with their acidic moieties predicted to interact with the basic, Gln298, Lys 301 and Arg305 cluster purple). D. Overlay of the binding poses of **F2** and **F29**, whose combined structure resembles losartan (**5**).

This assessment indicated all group 1, 2 and some group 3 fragments preferentially orientated their phenyl rings inside the aromatic‐hydrophobic Val4 binding pocket. From here the acidic moieties generally positioned themselves in close proximity to the Gln298, Lys301 and Arg305 cluster. From this orientation, the 4‐hydroxyl moiety of the most active fragment **F5** (Figure 5A), as well as **F7**–**F9**, were predicted to form two additional H‐bond with Asn233 and Tyr248 located within the hydrophobic pocket. The *ortho* substituted fragments orientated their substituents in the direction of the Asp5 binding region, with the pyridine moiety of **F10** (Figure 5B) was predicted to form an electrostatic interaction with Lys229, which was unique amongst the docked fragments. In addition to pushing the tetrazole moiety closer toward the Gln298, Lys 301, Arg305 cluster, the benzylic carbon of **F16** (Figure 5C) and **F17** affords extra conformational flexibility, allowing the tetrazole to sample more of this basic binding region. In contrast to **F16** and **F17**, the carboxylic acid moieties of mandelic acid analogues (**F20**–**F24**) interacted only with Arg305.

From a stereochemical point of view, the alpha hydroxyl moiety of the *R*‐ and *S*‐ isomers were predicted to interact with Arg305 and Asn264, respectively, with no clear preference in the docking energies or scores (Figure S2A). Like the group 1 and 2 fragments, the aromatic portion of isoindolinone containing **F26** was predicted to bind in the Val4 pocket, with the lactam carbonyl and acid moiety predicted to interact with Arg305 and Gln298, respectively (Figure S2B). While the aromatic portion of **F27** (Figure S2C) and **F28** were predicted to bind in the vicinity of the Val4 pocket, the acid moiety of both the *R‐* and *S*‐ isomers of both fragments orientated in a similar position to the pyridine substituent of **F10**, forming a series of salt bridges with Lys229, Asn264 and Asn233. Finally, **F29** had a unique predicted pose amongst the binding fragments, forming interactions with Arg305 and Asn308 in the Glu2 binding region.

**Figure 6:**
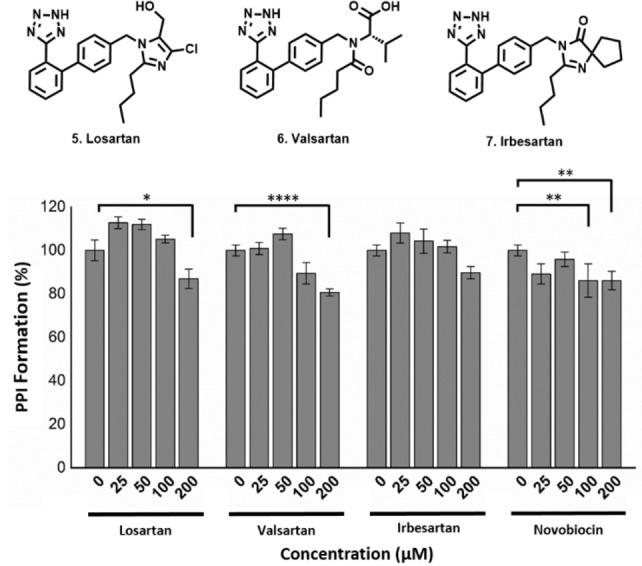
Solid phase PPI ELISA assay, between HOP TPR2A domain and the HSP90 *C*‐terminal domain. Losartan displayed a mild yet statistically significant stabilizing effect on the PPI at 25 μM. Both losartan and valsartan displayed statistically significant PPI inhibitory activity at 200 μM. * p<.05; ** p<.01; *** p<.001; **** p<.0001.

A structural overlay of the docked poses of group 1 and 2 fragments with that of **F29** (Figure 5D), resembled the *ortho* biphenyl tetrazole losartan (**5**). We reasoned that **5** alongside valsartan (**6**) and irbesartan (**7**) might provide additional insight into future elaboration campaigns. The predicted binding pose of all three compounds was not in close agreement with that of our fragments (Figure S3), which was possibly due to the bulky butyl side chains of the ‘sartans’, altering the accessibility to the binding site predicted for the smaller fragments. However, TSA indicated that the presence of all three compounds resulted in a statistically significant shift in thermal stability of TPR2A, likely because of protein binding (Figure 3A and C). Similarly, all three compounds displayed a moderate and dose‐dependent disruption of the target PPI (Figure 6) and were important in confirming the promise of elaborated *ortho* substituted phenyl tetrazoles as HOP‐HSP90 PPI inhibitors.

## Conclusion

The ability to rapidly detect electrostatic interactions between protein targets and small molecules through native MS is becoming an increasingly useful tool in contemporary drug discovery. Furthermore, the inherent bias toward identifying compounds with preferable physicochemical properties offers additional value in this approach.

Together, the sensitivity, speed, and low sample consumption of native MS, particularly when coupled with nano‐ESI in a multiwell format, makes a compelling case for greater application of native MS‐based screens as an orthogonal technique for the identification of weakly binding fragments as well as small molecules capable of binding to PPI interfaces. Despite this, there are only a limited number of reports pertaining to its application to FBDD and PPI drug discovery, respectively.

In this study, our nano‐ESI, native MS screening strategy, identified a cohort of buffer soluble fragments, which bind to the TPR2AB domain of HOP. In addition to a comparatively simple experimental set up, each binding experiment required only four minutes of data acquisition. This compares favourably to the laborious handling or the lengthy experimental times for many other fragment screening techniques. Orthogonal thermal shift assay confirmed that a small subset of these fragments bound to the single domain of TPR2A, while three of these fragments were confirmed disrupt the target TPR2A‐HSP90 *C*‐terminal PPI, thus providing an important validation of this approach. *In‐silico* evaluation of the binding fragments at the TPR2A‐MEEVD interface provided structural insight into their binding modes, which alongside the structural information of binding and non‐binding fragments, illuminated potential fragment elaboration strategies. Given the resemblance of the peptidomimetic losartan to a postulated elaborated fragment, we assessed three ‘sartans’ for their PPI inhibitory activity, where they were found to possess weak albeit dose‐dependent PPI inhibitory activity. While this activity can be considered low, previously reported small molecules have generally only been shown to disrupt MEEVD binding and not formal PPI disruption. Furthermore, and most importantly, this result confirmed the potential of *ortho* substituted phenyl tetrazoles as scaffolds for inhibiting this PPI. As such, this will form the basis of a refined fragment elaboration strategy, which will combine promising structural features of binding fragments in order to enhance PPI inhibitory activity.

## Experimental Section

**Chemicals and materials**: Small molecules used in this study, were purchased from Key Organics, Sigma Aldrich and Alfa Aesar (purity ≥95 %). LCMS grade dimethyl sulfoxide (DMSO) and LCMS grade ammonium acetate (NH_4_OAc) were purchased from Sigma Aldrich.

**Protein expression and purification**: *E. coli* BL21(DE3) cells were transformed with the pGEX‐3X‐1400 plasmid containing the coding region for the TPR2AB region (residues 208–543) from murine HOP (which shares 98 % amino acid sequence identity with human HOP, and has been shown to interact effectively with human HSP90).[^32^, ^33^] The production of GST‐TPR2AB protein was induced by addition of 1 mM isopropylthio‐β‐galactoside (IPTG) for 3 hours at 37 °C and GST‐TPR2AB purified by native GSH‐affinity chromatography.^[34]^ The GST tag was removed to yield the untagged TPR2AB domain using the Factor Xa Cleavage Capture Kit (Merck Millipore) according to the manufacturer's instructions. The stages of protein purification and proteolytic cleavage of the GST tag was monitored by SDS‐PAGE and Western blot analysis.^[34]^ The average yield of TPR2AB was 0.70±0.05 mg/L.

**Sample preparation**: Samples of TPR2AB or TPR2A were buffer exchanged into 100 mM NH_4_OAc using Zeba Spin Desalting Column (Thermo Fisher Scientific) prior to MS analysis. Concentration of TPR2AB screening solution was adjusted to 20 μM with 100 mM NH_4_OAc buffer.

50 mM stock solutions of the fragments were prepared in LCMS grade DMSO. 2.5 mM screening solutions (5 % DMSO) were prepared by diluting 5 μl aliquots of the DMSO stocks with 95 μl of 100 mM NH_4_OAc buffer.

For fragment binding analysis against TPR2AB, 6 μl and 2 μl aliquots of the TPR2AB and fragment screening solutions respectively were mixed with a further 12 μl of 100 mM NH_4_OAc buffer, to a final analysis concentration of 6 μM TPR2AB, 250 μM fragment, 0.5 % DMSO. Samples were prepared in batches of 6, and held at 4 °C prior to MS analysis. For MS analysis against TPR2A, the mixture was adjusted to 2 μl of TPR2A, 15 μl of 100 mM NH_4_OAc buffer and 3 μl of fragment stock to a final analysis concentration of 6 μM TPR2AB, 125 μM fragment, 0.25 % DMSO.

**Native MS analysis**: Native MS and IM‐MS data were obtained on both a Synapt‐G2 Q‐TOF (Waters). Ionisation was achieved using a NanoMate nESI infusion robot (TriVersa), sampling from a 96‐well plate. All experiments were conducted under the same native MS conditions, i. e. nanoelectrospray voltage of 1.54 kV, cone voltage 100 V, trap voltage of 5 V and a source temperature of 60 °C, while backing pressure was adjusted to 4.0 mbar. Final spectra were the sum of 240 scans collected over four minutes. MS data were processed using MassLynx v4.0 (Waters).

HSP90 C‐TPR2A PPI inhibition and *in‐silico* assessment were conducted using previously reported methods.^[26]^

**Thermal shift assay**: The TPR2A protein was diluted to a final concentration of 20 μM in buffer (50 mM Tris‐HCl, pH 7.5, 100 mM NaCl) containing 20X SYPRO Orange dye (from 100X stock, Merck S5692). The thermal stability of the protein was monitored in the presence and absence of ligands (Ac−MEEVD−OH peptide, drugs, or fragments). Vehicle, buffer, and ligand only controls were included and consistently showed no signal. Reactions were equilibrated at 25 °C and subjected to thermal scanning from 25 °C to 95 °C (0.5 °C/min) in a Bio‐Rad CFX96 qPCR machine with readings collected every minute in the FRET channel. The melting temperature (Tm) of the TPR2A protein was calculated from the change in fluorescence over time (−dRFU/dt).

## Conflict of interest

The authors declare no conflict of interest.

1

## Biographical Information

*Clinton Veale is an Associate Professor or Organic Chemistry at the University of Cape Town and a Royal Society‐ AAS Future Leaders (FLAIR) Fellow. Prior to this, Clint was a Claude Leon Post‐Doctoral Research Fellow at Stellenbosch University, a lecturer in Pharmaceutical Chemistry at Rhodes University and a Senior Lecturer in Organic Chemistry at the University of KwaZulu‐Natal. His research interests lie at the interface of chemistry and biology and combines synthetic organic chemistry and native mass spectrometry methodology to develop small molecule inhibitors of challenging targets*.

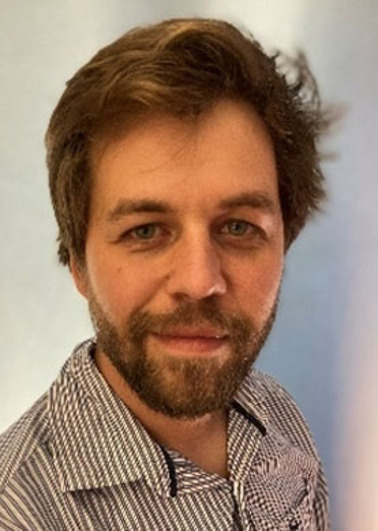

## Supporting information

As a service to our authors and readers, this journal provides supporting information supplied by the authors. Such materials are peer reviewed and may be re‐organized for online delivery, but are not copy‐edited or typeset. Technical support issues arising from supporting information (other than missing files) should be addressed to the authors.

Supporting Information

## Data Availability

The data that support the findings of this study are available from the corresponding author upon reasonable request.
