# Supplementary material for: Native Mass Spectrometry‐Guided Screening Identifies Hit Fragments for HOP‐HSP90 PPI Inhibition
Source: Chembiochem. 2022 Sep 20;23(21):e202200322. doi: 10.1002/cbic.202200322 (PMC9826382; doi:10.1002/cbic.202200322)
Supplement: Supplementary file 1 — Supporting Information [file CBIC-23-0-s001.pdf]

ChemBioChem

Supporting Information

Native Mass Spectrometry-Guided Screening Identifies Hit Fragments for HOP-HSP90 PPI Inhibition**

Michaelone C. Vaaltyn, Maria Mateos-Jimenez, Ronel Müller, C. Logan Mackay, Adrienne L. Edkins, David J. Clarke, and Clinton G. L. Veale*

Mass Spectra of binding fragments

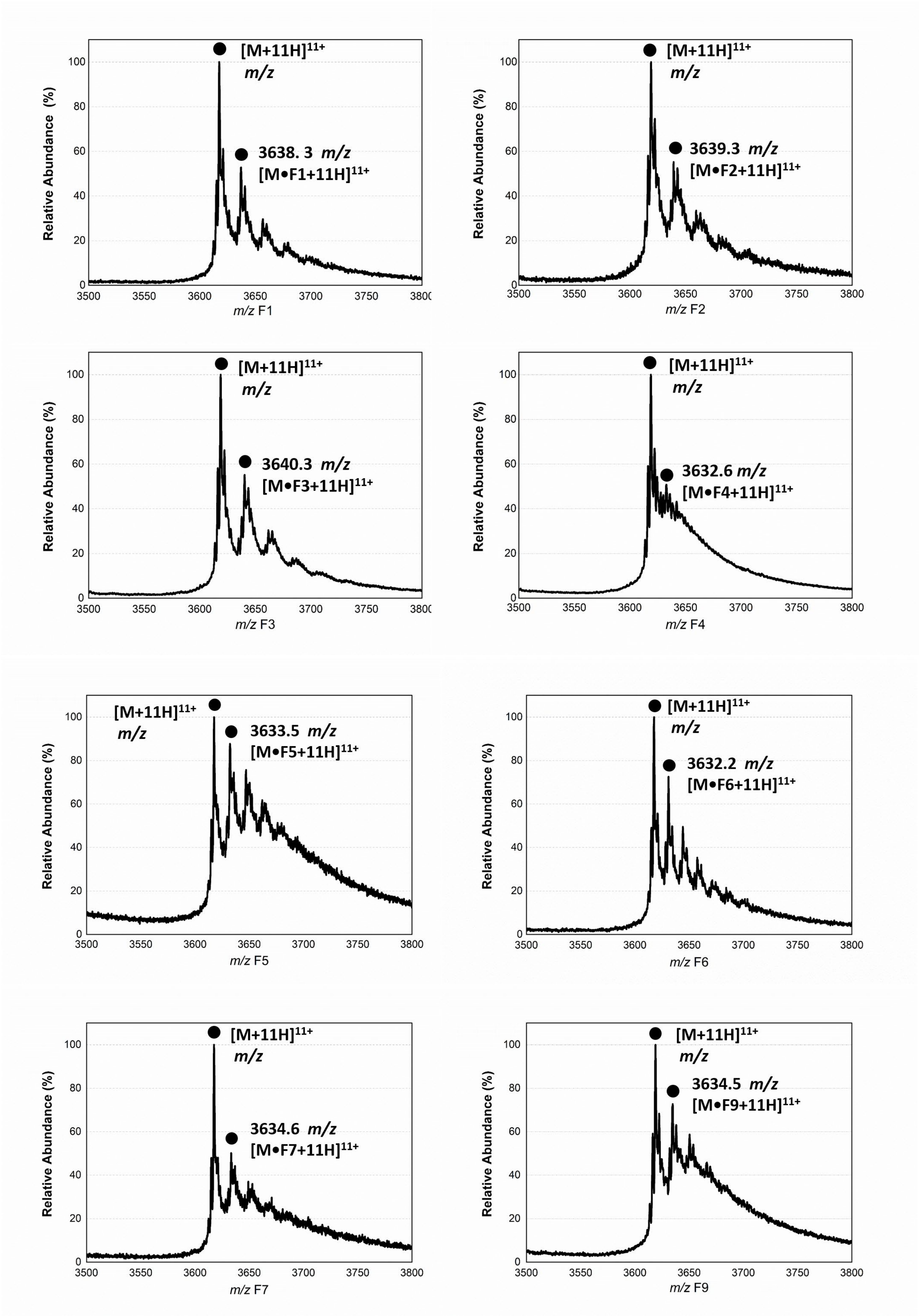

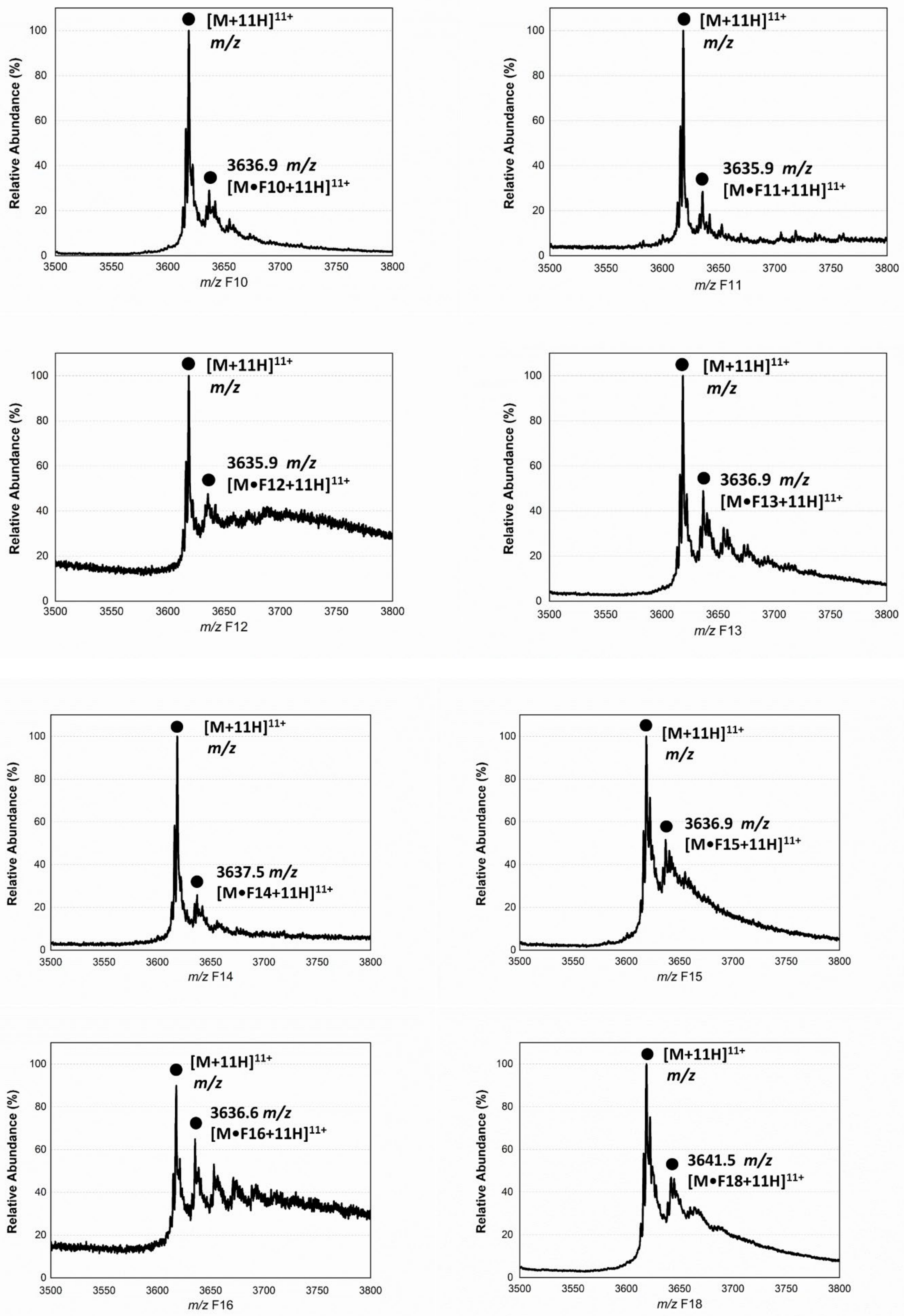
[M+11H]11+ m/z
3636.9 m/z [M•F10+11H]11+
Relative Abundance (%)
m/z F10
[M+11H]11+ m/z
3635.9 m/z [M•F11+11H]11+
Relative Abundance (%)
m/z F11
[M+11H]11+ m/z
3635.9 m/z [M•F12+11H]11+
Relative Abundance (%)
m/z F12
[M+11H]11+ m/z
3636.9 m/z [M•F13+11H]11+
Relative Abundance (%)
m/z F13
[M+11H]11+ m/z
3637.5 m/z [M•F14+11H]11+
Relative Abundance (%)
m/z F14
[M+11H]11+ m/z
3636.9 m/z [M•F15+11H]11+
Relative Abundance (%)
m/z F15
[M+11H]11+ m/z
3636.6 m/z [M•F16+11H]11+
Relative Abundance (%)
m/z F16
[M+11H]11+ m/z
3641.5 m/z [M•F18+11H]11+
Relative Abundance (%)
m/z F18

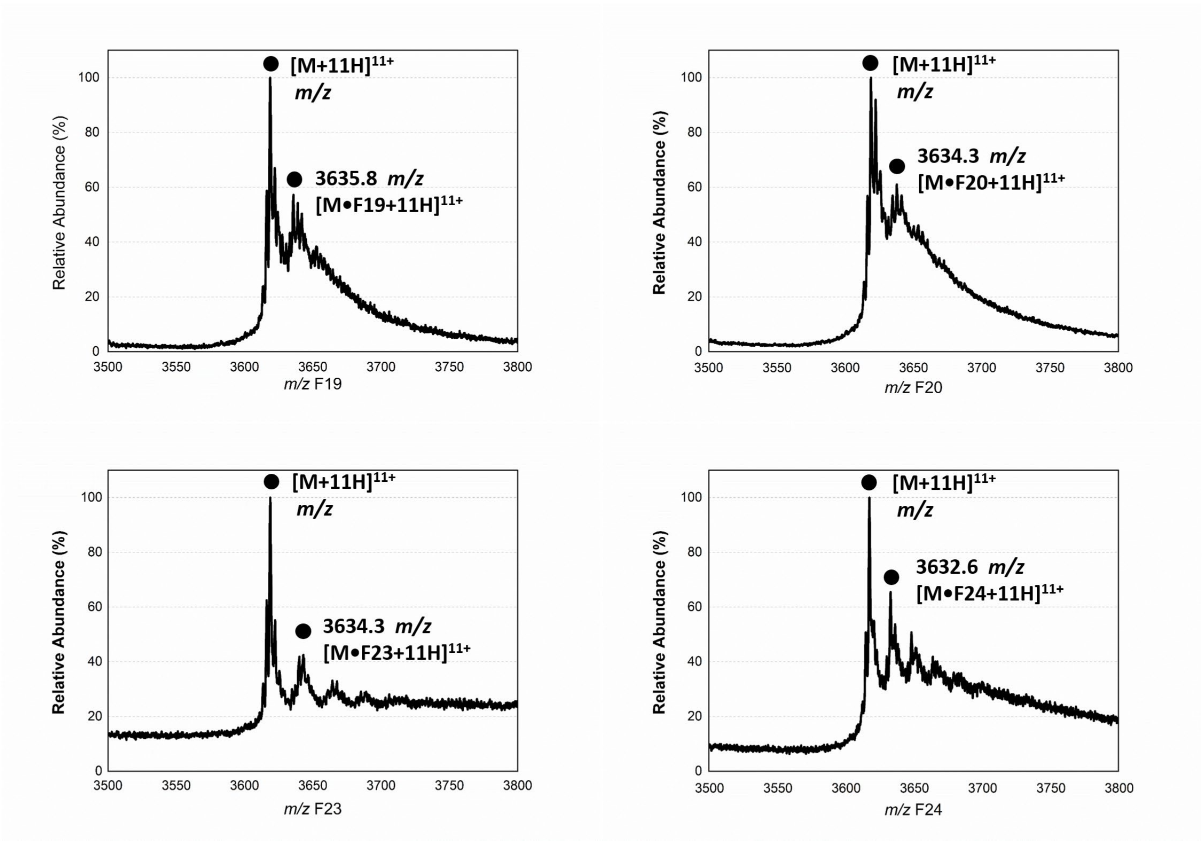
[M+11H]11+
m/z
3635.8 m/z
[M•F19+11H]11+
Relative Abundance (%)
m/z F19
[M+11H]11+
m/z
3634.3 m/z
[M•F20+11H]11+
Relative Abundance (%)
m/z F20
[M+11H]11+
m/z
3634.3 m/z
[M•F23+11H]11+
Relative Abundance (%)
m/z F23
[M+11H]11+
m/z
3632.6 m/z
[M•F24+11H]11+
Relative Abundance (%)
m/z F24

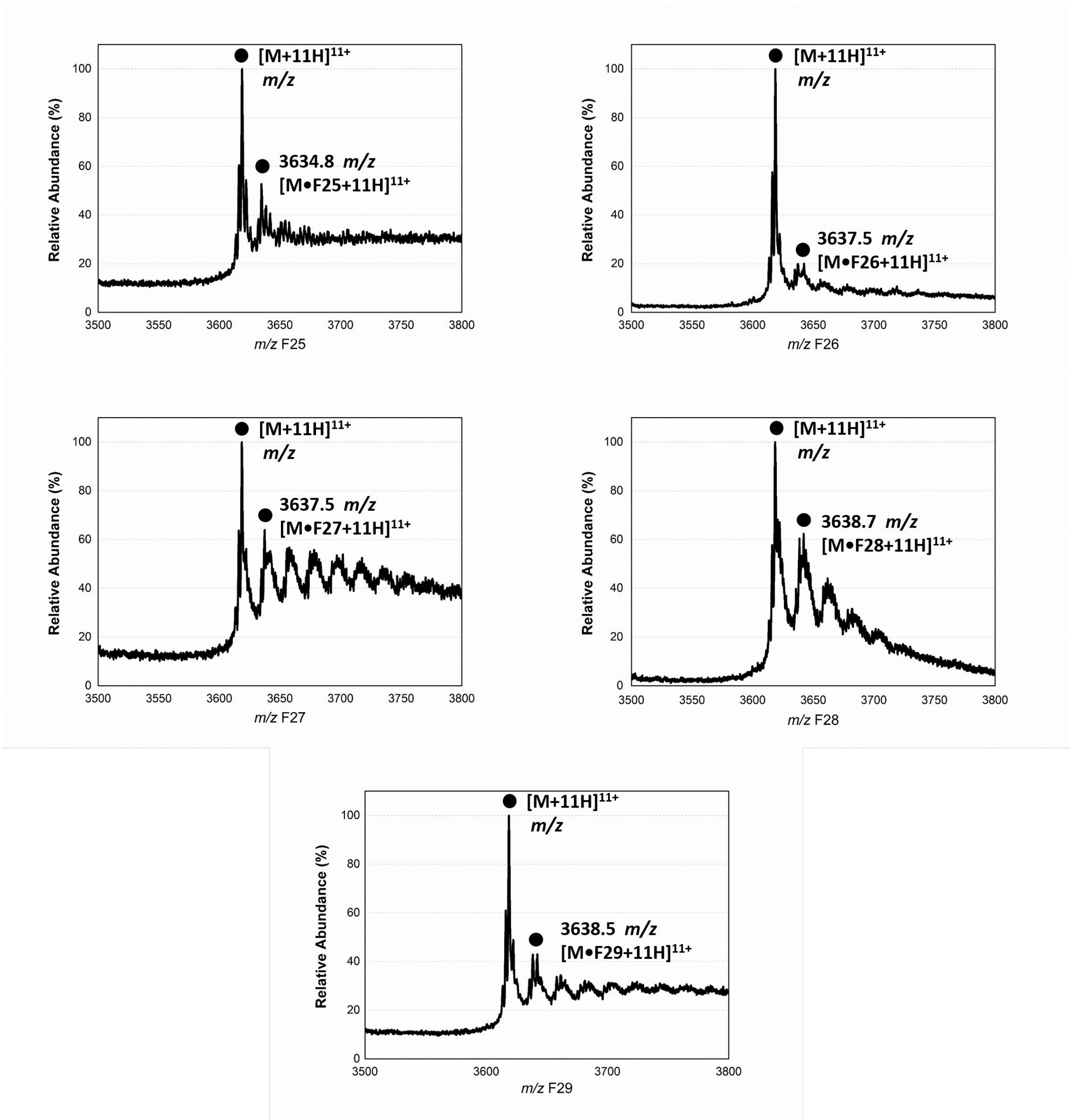
[M+11H]11+ m/z
3634.8 m/z [M•F25+11H]11+
m/z F25
[M+11H]11+ m/z
3637.5 m/z [M•F26+11H]11+
m/z F26
[M+11H]11+ m/z
3637.5 m/z [M•F27+11H]11+
m/z F27
[M+11H]11+ m/z
3638.7 m/z [M•F28+11H]11+
m/z F28
[M+11H]11+ m/z
3638.5 m/z [M•F29+11H]11+
m/z F29
Relative Abundance (%)

Table S1: Non-binding fragments

Compound number	Structure
S1	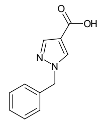
S2	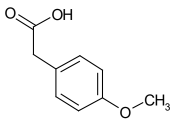
S3	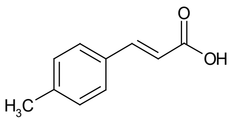
S4	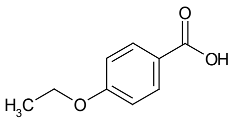
S5	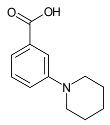
S6	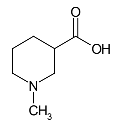
S7	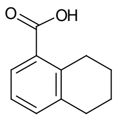
S8	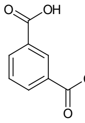
S9	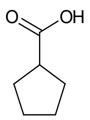

S10	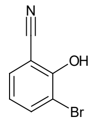
S11	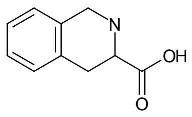
S12	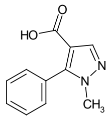
S13	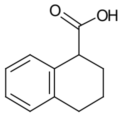
S14	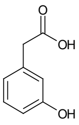
S15	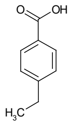
S16	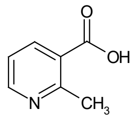
S17	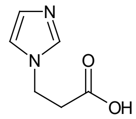
S18	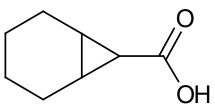
S19	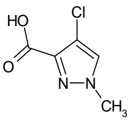
S20	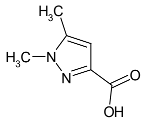

S21	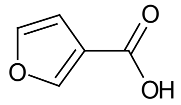
S22	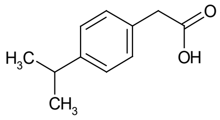
S23	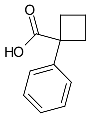
S24	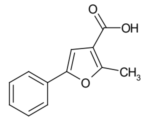
S25	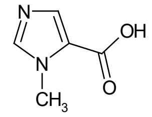
S26	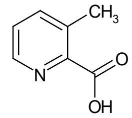
S27	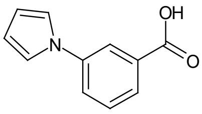
S28	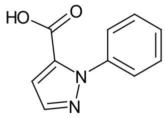
S29	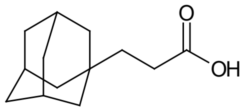
S30	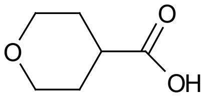
S31	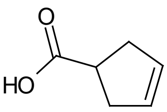

S32	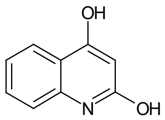
S33	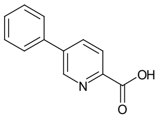
S34	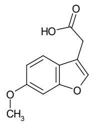
S35	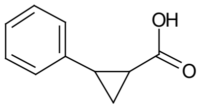
S36	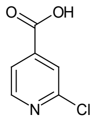
S37	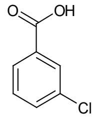
S38	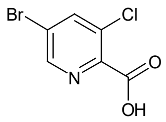
S39	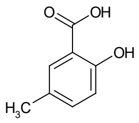
S40	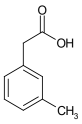
S41	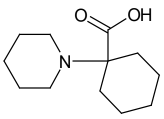
S42	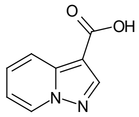

S43	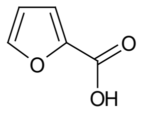
S44	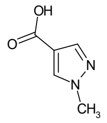
S45	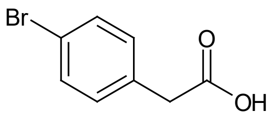
S46	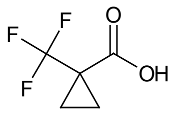
S47	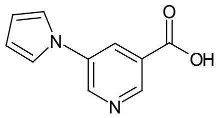
S48	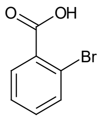
S49	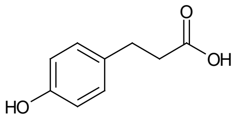
S50	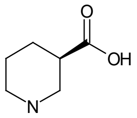
S51	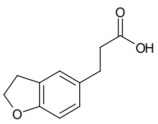
S52	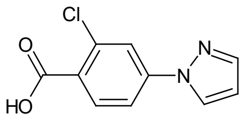
S53	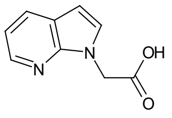

S54	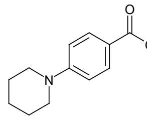
S55	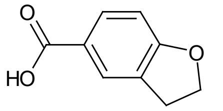
S56	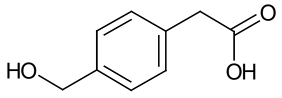
S57	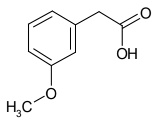
S58	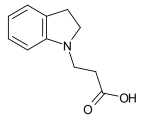
S59	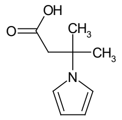
S60	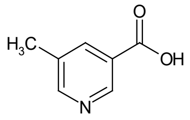
S61	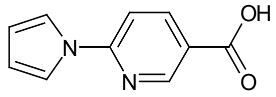
S62	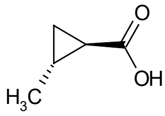
S63	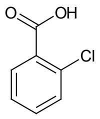
S64	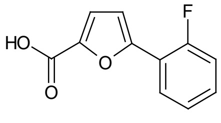

S65

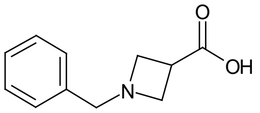

S66

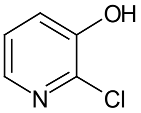

S67

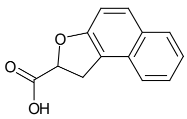

S68

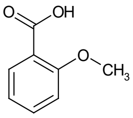

S69

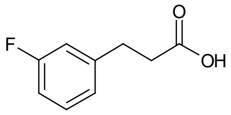

S70

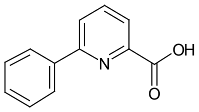

S71

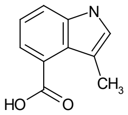

S72

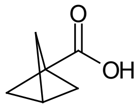

S73

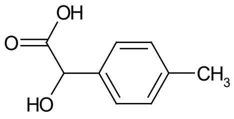

S74

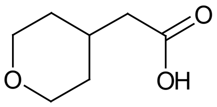

S75

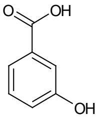

S76	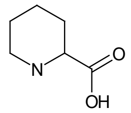
S77	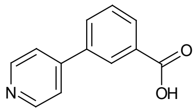
S78	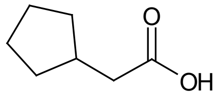
S79	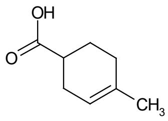
S80	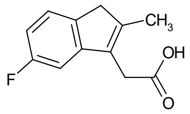
S81	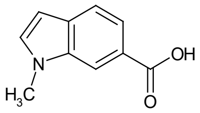
S82	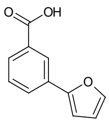
S83	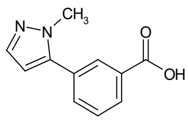
S84	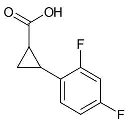
S85	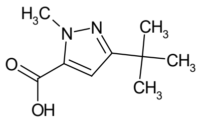
S86	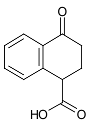

S87

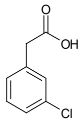

S88

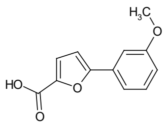

S89

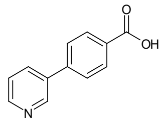

S90

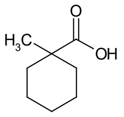

S91

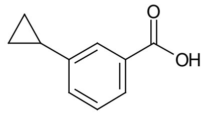

S92

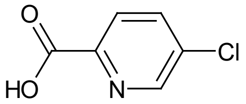

S93

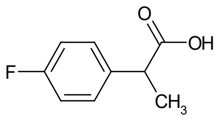

S94

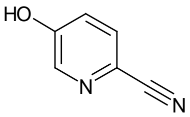

S95

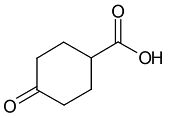

S96

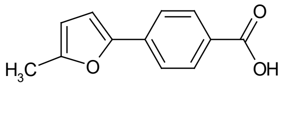

S97

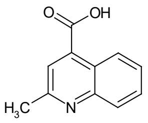

S98	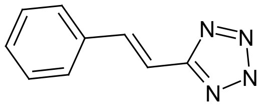
S99	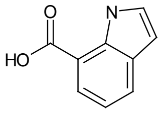
S100	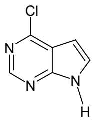
S101	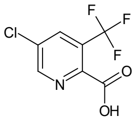
S102	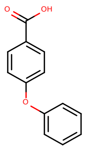
S103	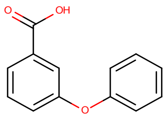
S104	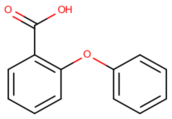

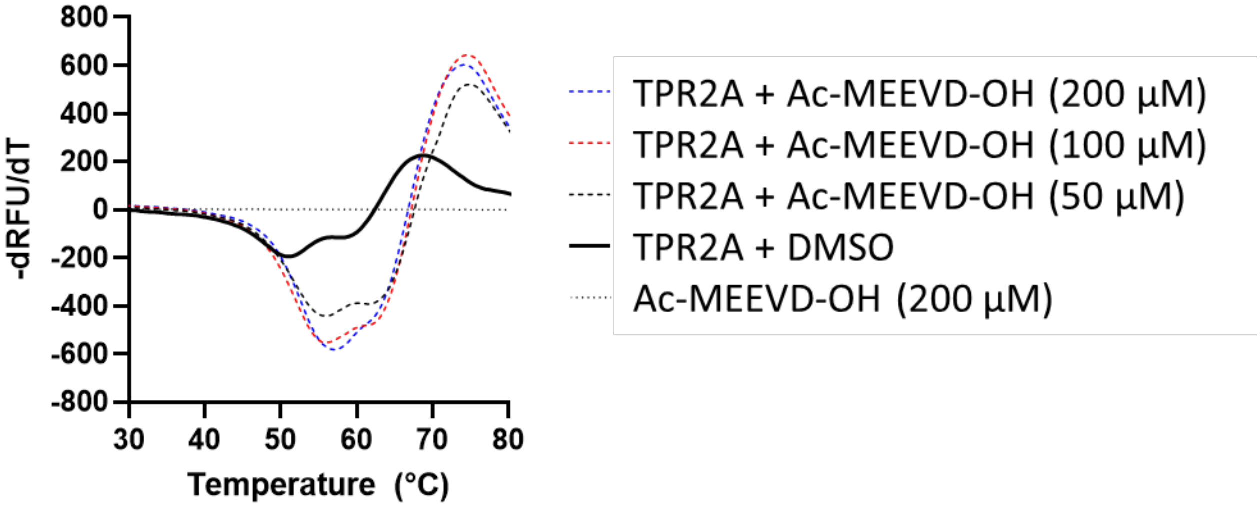

Figure S1. Melt curves for the TPR2A protein in the absence (solid black line) and presence (dashed or coloured lines) of Ac-MEEVD-OH. The shift in the location of the highest negative peak to a higher temperature indicates stabilisation of the protein in the presence of ligands.

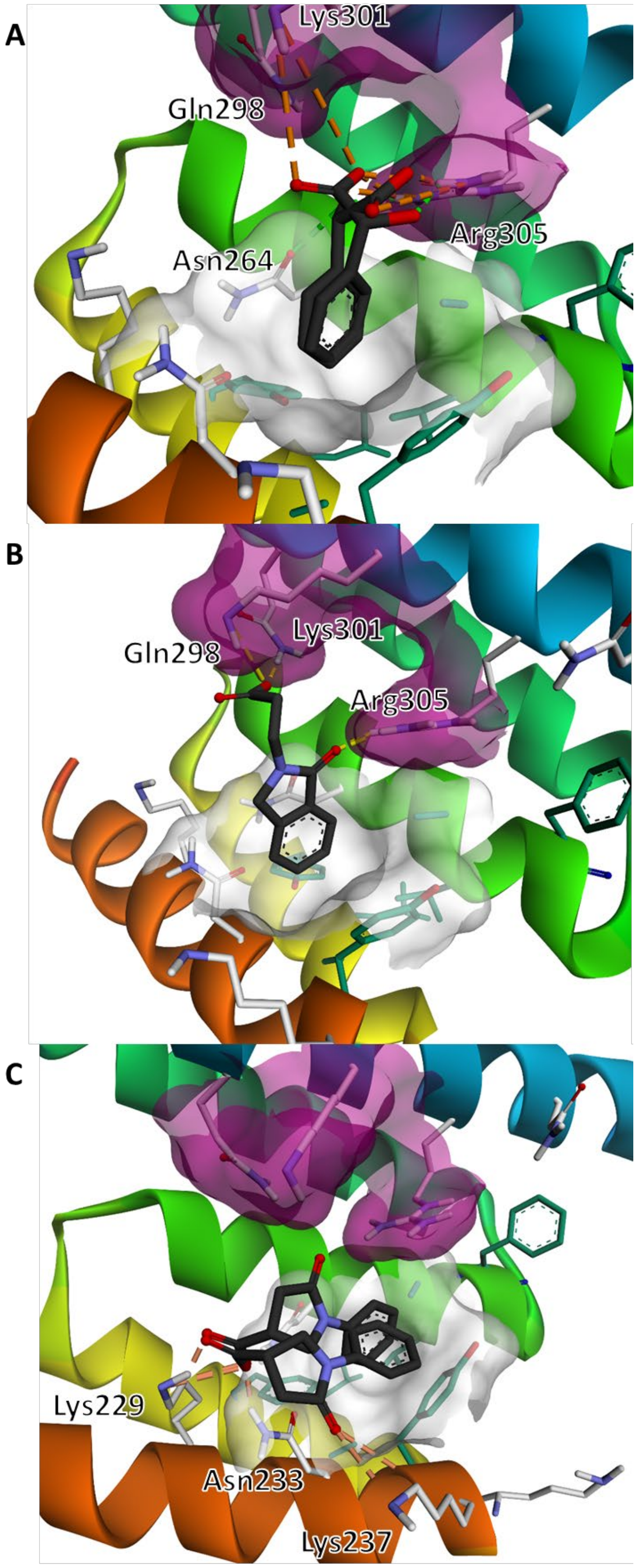

Figure S2A. Predicted binding pose of the *R* and *S* isomer of **F24**. **B**. Predicted binding pose of **F26**. **C**. Predicted binding pose of the *R* and *S* isomer of **F27**

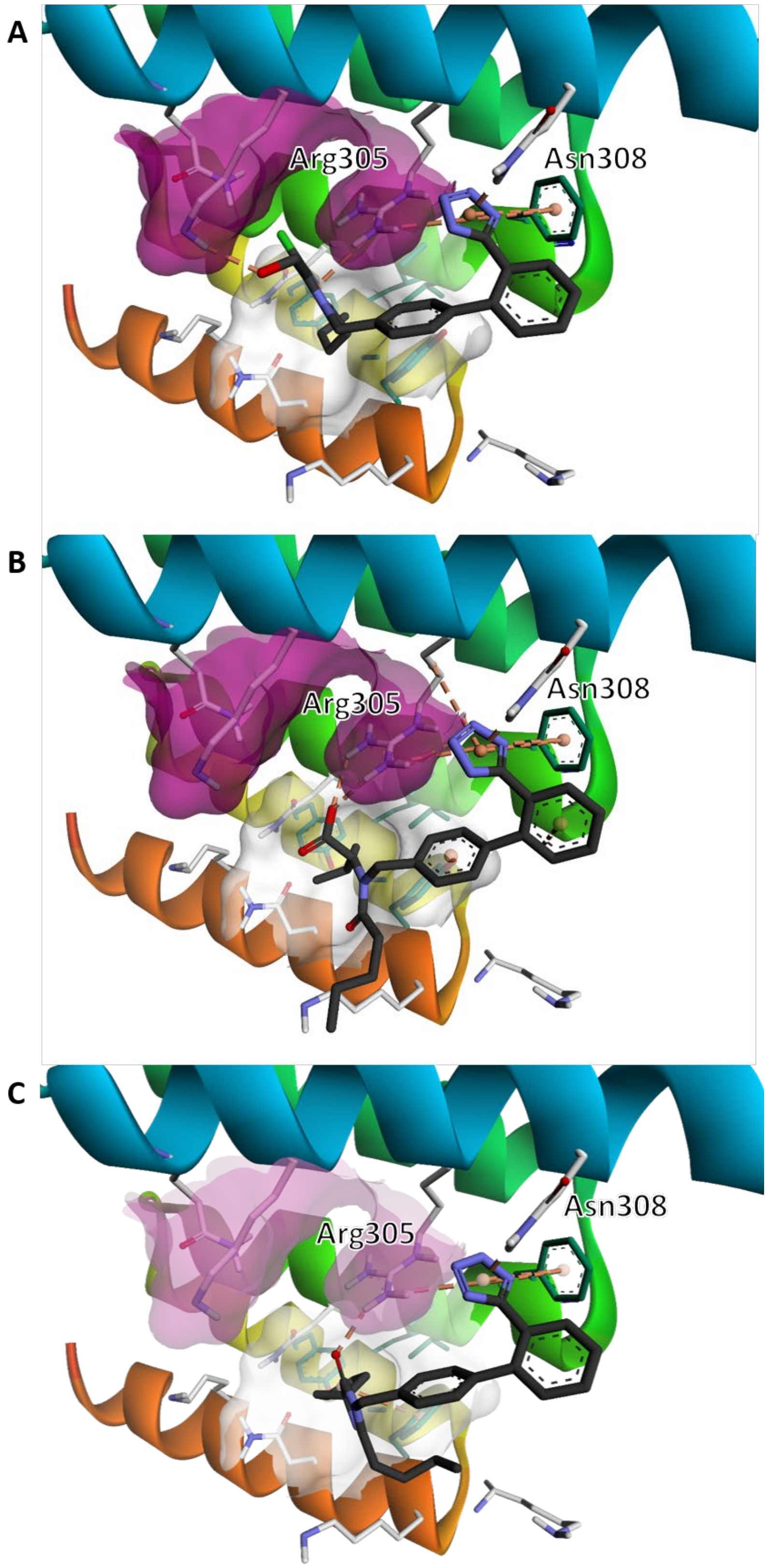

Figure S3. Predicted binding pose of Losartan (**A**), Valsartan (**B**) and Irbesartan (**C**). In contrast to the group 1 and 2 fragments, the tetrazole preferentially interacted at a cleft formed between Arg305 and Asn308
